# Development of Ultraviolet-Shielding Bamboo/Silk Fibroin Hybrid Films with Good Mechanical Properties: A Proof Study on Human Keratinocyte Cells

**DOI:** 10.3390/polym16162244

**Published:** 2024-08-07

**Authors:** Silvia Bittolo Bon, Valeria Libera, Maria Rachele Ceccarini, Rocco Malaspina, Michela Codini, Luca Valentini

**Affiliations:** 1 Dipartimento di Fisica e Geologia, Università degli Studi di Perugia, Via A. Pascoli, 06123 Perugia, Italy; silvia.bittolo@gmail.com (S.B.B.); valeria.libera@unipg.it (V.L.); rocco.malaspina@dottorandi.unipg.it (R.M.); 2 Department of Pharmaceutical Science, University of Perugia, 06123 Perugia, Italy; mariarachele.ceccarini@unipg.it (M.R.C.); michela.codini@unipg.it (M.C.); 3 Civil and Environmental Engineering Department and INSTM Research Unit, University of Perugia, Strada di Pentima 8, 05100 Terni, Italy

**Keywords:** silk fibroin, bamboo, UV shielding, mechanical properties, cells

## Abstract

In this study, we report the preparation and characterization of water-stable films with UV-shielding and good mechanical properties, exploiting the synergistic effect of regenerated silk fibroin and bamboo-derived cellulose. Silk fibroin (SF)/bamboo (B) hybrid films are achieved by solubilizing both silk and bamboo fibers in formic acid with added CaCl_2_. Infrared spectroscopy indicates that SF, when combined with bamboo, undergoes a conformational transition, providing evidence of an increase in SF crystallinity. Exploiting the intrinsic absorption of SF in the ultraviolet region, UV–Vis spectroscopy was used to assess the glass transition temperature (T_g_) of SF/B films, showing a decrease in T_g_ by increasing the SF content. The addition of 10 wt% SF to the B matrix improved the elastic modulus by about 10% while conserving the strain at break with respect to the neat B films, increasing the UV shielding properties, while water absorption suggested the material’s hydrophilic and swelling capacity even after one month. The hybrid films showed, under solar irradiation, a photoprotective behavior on keratinocyte human cells by increasing cellular viability. These findings may find potential applications in functional fabrics.

## 1. Introduction

Silk fibers spun by silkworms are, among all natural polymers, those with the best biological and mechanical properties [1,2,3]. The intrinsic high mechanical strength and elongation at break of silk, along with its biocompatibility [4,5,6,7], can be potentially used for multifunctional high-performance textiles. The main drawback is the fact that spiders and silkworms produce silk for their biological needs, and, thus, upscaling production is extremely challenging as the variability in the climatic conditions where silkworms take their food to spin these fibers creates an intrinsic variation in the mechanical properties of the final product [8,9,10,11]. Currently, the use of regenerated silk fibroin (SF) obtained from natural fibers is a viable method for the large-scale production of natural polymers. The principal bottleneck of this approach is the variability in the properties depending, for example, on the humidity that plasticizes the silk, reducing the tensile strength, the degumming process, which affects conformational transition, making the fibers soluble in water [12,13,14,15], and the dissolving process, which, finally, destroys the original multiscale fibrillar structure, increasing the brittleness [16].

Polymer blending is a practical approach that is currently adopted to obtain SF-based composites with desired properties [17,18]. Among all biopolymers, SF films’ properties can be improved by blending SF with cellulose—a biocompatible, low-cost, and abundant natural polymer [19,20,21]. SF with UV-absorbing properties can be obtained by tuning the conformational transition [22]. Bamboo is a lignocellulosic green raw material that, for its fast growth rate, abundance, and outstanding mechanical properties, is currently widely used, from building applications to the textile sector [23,24,25,26].

It is well known that solar UV radiation is the most important risk factor for skin diseases, with UVA (320–400 nm) and UVB (280–320 nm) components [26]. UV-blocking effects are based on UV absorption and UV-scattering materials [27]. Thus, the development of novel materials protecting one from ultraviolet (UV) rays represents the frontier for these applications. Since cellulose lacks UV absorption properties despite its intrinsic mechanical properties, combining bamboo-derived cellulose with the natural photoprotective properties of SF could be a viable approach to developing bio-based photoprotective films. In the past, we realized that sunscreens prevented the damage produced by UV radiation on epidermal cells. The sustainable nature of the proposed materials as well as the easy processing method may be considered valid alternatives to the metal oxide additives currently used in cosmetics to block UV rays, which have the drawback of inducing the production of reactive oxygen species, stimulating oxidative stress in tissues.

## 2. Materials and Methods

### 2.1. Bamboo Solution Preparation

Commercial bamboo (B) yarns (supplied by a local farm) were used to produce films using a solvent-casting method: 200 mg of bamboo yarn was cut in small pieces and solubilized in 10 mL of formic acid (FA) through magnetic stirring while keeping the solution at 80 °C for 2 h (bamboo content in the FA solution: 20 mg/mL).

### 2.2. Silk Fibroin and Silk Fibroin/Bamboo Hybrid Solution Preparation

SF was previously extracted from *Bombyx mori* cocoons (supplied from a local farm) in hot deionized water and sodium bicarbonate (supplied by Sigma Aldrich, St. Louis, MO, USA): 5 silk cocoons were soaked in 200 mL of boiling deionized water containing 5 g of sodium bicarbonate for 45 min; the silk fibers were then rinsed with deionized water; and the complete treatment was repeated two times to separate SF from all the sericin component of the silk cocoon. SF was left at room temperature until its complete drying. SF was solubilized in formic acid (purity 98%, supplied by Sigma Aldrich) and calcium chloride (CaCl_2,_ Sigma Aldrich) at room temperature for 5 min using magnetic stirring: 5 mL of FA with CaCl_2_ was used to dissolve 700 mg of SF, and the weight ratio between SF and CaCl_2_ was 70:30 (silk content in the FA solution: 140 mg/mL). Hybrid solutions were prepared mixing neat SF solution and B solution, with two different volume ratios considered: SF/B 10/90 and SF/B 50/50. The solutions were mixed at room temperature with magnetic stirring (mixing time: 5 min).

### 2.3. Silk Fibroin and Silk Fibroin/Bamboo Hybrid Film Preparation

The SF solution was poured in an uncovered Petri dish, and the FA was left to evaporate at room temperature for 12 h and annealed at 40 °C for 2 h to achieve complete solvent removal. Hybrid films were obtained by casting the solutions in Petri dishes with a diameter of 5 cm. The Petri dishes were left open at room temperature to allow complete solvent evaporation and then annealed at 40 °C for 2 h.

### 2.4. FTIR Characterization

Infrared spectra were recorded in transmission mode using a Fourier transform spectrometer from Jasco (Oklahoma City, OK, USA) (model FTIR 615). The spectra recorded in the 4000−400 cm^−1^ spectral range with a resolution of 2 cm^−1^ were averaged from 300 scans. To estimate the different components of amide I spectral profiles for the samples containing SF, a curve-fitting procedure was employed. Each component was assigned a Gaussian line shape, a full width at half height (FWHH) fixed at 20 cm^−1^, and the weight was determined without constraints.

### 2.5. Degradation and Mechanical Characterization

To investigate degradation, SF and SF/B films (circular shape with a diameter of 5 cm and thickness of 300 μm) were immersed at room temperature in 10 mL of phosphate-buffered saline (PBS) with a pH of 7.4. At a designated time, the samples were washed with distilled water, dried in a desiccator, and weighed to estimate the weight variation.

A tensile testing machine (Lloyd Instr. LR30 K, Fareham, UK) was used to measure the mechanical properties of the films. The films were cut to have rectangular-shaped samples with the following dimensions: 1 cm × 3 cm × 300 µm. The samples were stretched with a strain rate of 5 mm/min using a 500 N load cell. The mechanical characteristics (i.e., Young’s modulus, tensile strength, and strain at break) were then calculated from the stress–strain curves.

### 2.6. Optical and Thermal Characterization

UV–Visible (UV–Vis) absorption measurements were performed using a Jasco V-570 spectrophotometer. A 0.01 mm path-length quartz cuvette was used in order to obtain the maximum signal-to-noise ratio. Each spectrum was collected in the range from 190 to 800 nm, with a scan speed of 100 nm/min. A Peltier temperature controller was used to change the temperature from 24 °C to 100 °C. To obtain information on the glass transition temperature (Tg), UV−Vis spectroscopy was employed to observe changes in the absorption spectrum due to the structural reorganization of polymers when heated above the Tg.

This property was quantified by the deviation metric of the temperature (DM_T_), defined as follows [28,29,30]:

$$DM_{T}=\sum_{\;\lambda_{min}}^{\lambda_{max}\;}{\left[I_{RT}\left(\lambda \right)-I_{T}\left(\lambda \right)\right]}^{2}$$

where I_RT_(λ) and I_T_(λ) are, respectively, the absorption intensity of the film at room temperature and at temperature T. The sum on the wavelength is taken on the full optical sweep around the absorption peak. The sum of a range of wavelengths affected by this variation was, for each sample, as follows: 250–350 nm, 400–500 nm, 350–500 nm, and 300–400 nm for the B, SF/B 10/90, SF/B 50/50, and SF, respectively.

### 2.7. Differential Scanning Calorimetry

Conventional differential scanning calorimetry (DSC) measurements were also performed with Perkin-Elmer Pyris (Waltham, MA, USA) on all the samples (10 mg), with a scan rate of 10 °C/min.

Measurements were taken in a temperature range from 40 °C to 300 °C. The instrument was calibrated using indium and zinc. To measure the film, an aluminum sample/reference pan was used.

### 2.8. Cell Culture

The HaCaT cell line comprises spontaneously transformed aneuploid immortal keratinocyte cells derived from adult human skin. Cells were purchased at passage level 46 from the Istituto Zooprofilattico Sperimentale della Lombardia e dell’Emilia Romagna (I.Z.S.L.E.R.) “Bruno Ubertini” (Brescia, Italy) and were used for experiments until 59 passages, routinely checking for their morphology. HaCat were grown in standard conditions, as previously described [31].

### 2.9. Irradiation and MTT Assay

During the experimental procedure, human keratinocyte cell lines were firstly trypsinized upon reaching 80% confluence, then harvested and counted using a trypan blue exclusion assay in a Bürker chamber. Cells were seeded using 35 mm dishes at three different concentrations based on the incubation time: 4 × 10^5^ cells/dish, 3 × 10^5^ cells/dish, and 2 × 10^5^ cells/dish, respectively, for 4, 24, and 48 h of incubation after constant and fixed irradiation (220 sec) under a 300 W Xenon light (ThermoOriel solar simulator model 69907 (Ljubljana, Slovenia)), as previously described in ref. [31]. All the cells were irradiated with the same condition: 1X pre-warmed PBS, which maintains cell viability during radiation exposure and does not absorb in the UV–Vis spectrum. The doses of radiation during 220 s exposure were 2.96 kJ/m^2^ (UVC), 6.95 kJ/m^2^ (UVB), 21.9 kJ/m^2^ (UVA), and 110 kJ/m2 (VIS-nIR), respectively. A negative control (CTR-) was kept under a polystyrene cover during solar simulation under the same conditions as the samples, which were covered, respectively, with different proportions of SF and B (SF, SF/B 50/50, SF/B 10/90, and B). A positive control (CTR +) was exposed to the solar simulator without any protection. The four patches with different proportions of SF and B were used, likewise, with a lid to cover the upper part of the dish, protecting the cell monolayer from the UV-nIR rays. Each dish was irradiated for 220 s, which corresponds approximately to an hour and a half of sun exposure to UV-B rays. Immediately after irradiation, the PBS was aspirated, and the DMEM complete medium was replaced. The HaCat cells were left for 4, 24, and 48 h, respectively, in an incubator with standard conditions. An MTT assay was performed to test cell viability. The stock solution (5 mg/mL) was freshly prepared and diluted 1:10 in a cell medium. The final concentration in each dish was 0.5 mg/mL. After 3 h in the incubator, the supernatant was completely eliminated. To lyse the cells, 1 mL of dimethyl sulfoxide (DMSO) was used for 30 min at 37 °C. The absorbance was measured in a 96-well plate at 540 nm by a spectrophotometer (Tecan Austria GmbH (Grödig, Austria), Model INFINITE 200 PRO). The experiments were performed in a biological triplicate. The % of viable cells was compared to CTR- using the following formula:

Viable cells (%) = (OD_sample/OD_CTR−) × 100

where OD is the optical density.

### 2.10. Statistical Analysis

In the statistical analysis for the comparison between multiple groups, a two-way ANOVA was conducted with significance thresholds of * *p* < 0.05, ** *p* < 0.01, *** *p* < 0.001, and **** *p* < 0.0001.

## 3. Results

**Figure 1:**
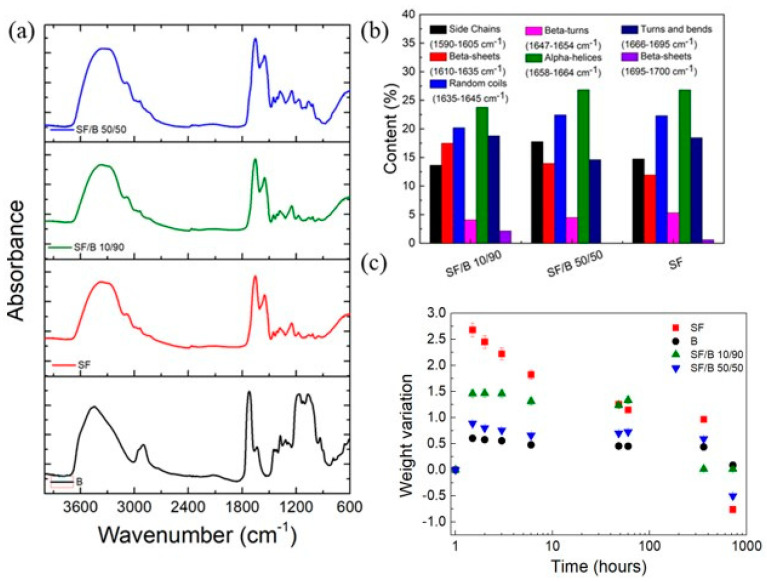
(**a**) FTIR spectra of the prepared films and (**b**) relative content of the secondary structures of SF obtained by curve-fitting the amide I region in the 1610–1700 cm^−1^ range according to that reported by Lu et al. [35]. (**c**) Degradation behaviors of B, SF, and SF/B films in PBS.

In this study, SF/B films were obtained via the dissolution of SF and bamboo fibers in FA. Thus, we could obtain information on the structure of both the bamboo and the regenerated silk films after the treatment in formic acid by means of FTIR. The infrared spectra of the B, SF, and SF/B samples are reported in Figure 1a. The bamboo sample showed characteristic absorption peaks for C−O−C, hemicellulose acetyl, C-H, and O-H cellulose stretching at 1160 cm^−1^, 1716 cm^−1^, 2902 cm^−1^, and 3334 cm^−1^ [32,33,34], respectively. The typical absorption peaks at 1606 and 1509 cm^−1^ of lignin [34] were not detected, indicating that lignin was removed from the bamboo after the FA treatment.

FTIR spectroscopy was also employed to analyze how the SF’s secondary structure was modified by the interaction with bamboo (Figure 1a). In the amide I (1600−1700 cm^−1^) and amide II (1500−1650 cm^−1^) FTIR regions [22], the absorption bands at 1640−1654 cm^−1^ and 1535−1545 cm^−1^ were assigned to a random-coil secondary structure, whereas the β-sheets gave rise to the bands at 1610−1630 cm^−1^ and 1510−1520 cm^−1^ [35,36,37], respectively. By fitting the amide I region with these components, we estimated the relative percentages of different secondary structures (β-sheets, turns, and random coils). As shown in Figure 1b, the addition of B produced an increase in the fraction of the peak components in the ranges of 1620–1625 (amide I) and 1697–1703 (amide I) related to the β-sheet structure. Specifically, the β-sheet content in the SF/B 10/90 and 50/50 films was found to be 18% and 13%, respectively, while an 11% content was estimated for the neat SF sample.

Then, we investigated the stability of the SF-based films in a PBS environment, since it was desirable to prove that they would be used in a biological environment (e.g., water) whilst avoiding their degradation [38,39]. All the samples (Figure 1c) showed an increase in their mass after a few hours of immersion, with this effect being more pronounced for the films containing SF. This was due to the water-trapping effect exerted by the Ca^2+^ ions dispersed into the fibroin. Ca^2+^ ions can trap water molecules due to the Ca ions’ capability to coordinate 6–8 water molecules via the oxygen atoms [40,41]. Moreover, the SF/B 10/90 film was stable even after 1 month, while an appreciable weight loss between 44 and 50% for the SF and SF/B 50/50 films was observed.

**Figure 2:**
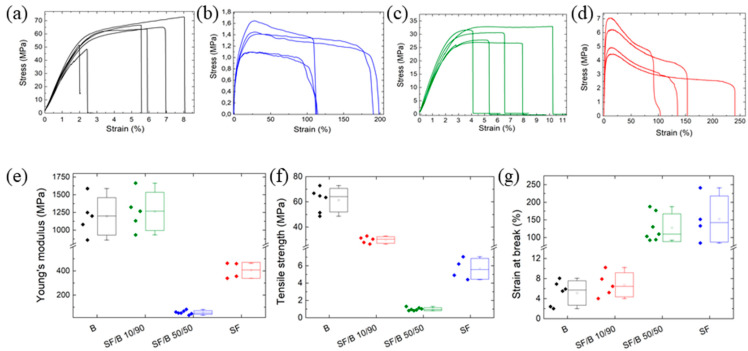
(**a**–**d**) Stress–strain curves of B, SF, SF/B 10/90, and SF/B 50/50 films, respectively. Influence of the SF content on the (**e**) Young modulus, (**f**) tensile strength, and (**g**) strain at break of all the analyzed samples.

Figure 2 shows the tensile tests for all the prepared samples. The mechanical characterization of the B (Figure 2a) and SF neat films (Figure 2b) shows very different behaviors: the bamboo films show high tensile strength values that are combined with a very low strain at break; the SF films, on the contrary, are characterized by a lower tensile strength and a high strain at break values. The influences of the amounts of added SF on the tensile strength, Young’s modulus, and strain at break of the B films were analyzed (Figure 2c,d), and they are reported in the box charts in Figure 2e–g. The pure B films presented a Young modulus of 1.2 ± 0.1 GPa (Figure 2e) and a strain at break (Figure 2g) of 6.2 ± 1.5%. When the concentration of SF increased from 10 to 50%, the Young modulus of the hybrid films and the strain at break varied from 1.3 ± 0.1 GPa to 55 ± 7 MPa and from 6.8 ± 1.2% to 127 ± 40%, respectively. The nanocomposite films achieved a maximal tensile strength of 29.8 ± 1.3 MPa when the addition of SF was 10% (Figure 2f). The improvement in the elastic modulus without a significant reduction in the strain at break were indicative of the fact that SF was dispersed homogeneously in the B films. The increase in the strain at break along with the tensile strength decrease as the SF was increased were due to the predominant mechanical behavior of the SF.

**Figure 3:**
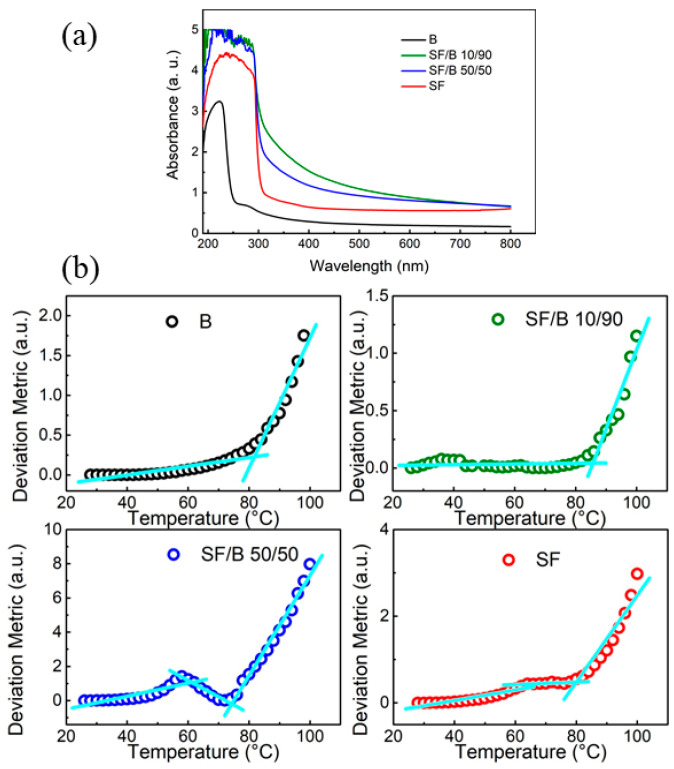
(**a**) UV–Vis spectra and (**b**) evolution of the deviation metric as a function of temperature of B, SF/B 10/90, SF/B 50/50, and SF films, respectively.

In Figure 3a, the UV–Vis absorbance spectra of the prepared samples are reported. The samples show absorptions in the UVC (100–280 nm), UVB (280–315 nm), and UVA (315–400 nm) regions, with these two last components being the most important for UV-shielding applications [42]. The results confirm that the absorption of the B film is evident in these regions but lower compared to samples containing SF. Bamboo’s UV-shielding ability is attributed to its lignin content [42]. The UV–Vis absorption spectra of the SF and SF/B films, on the contrary, indicate saturation around 280 nm, which is likely resulting from aromatic amino acids such as tyrosine, phenylalanine, and tryptophan, present along the SF chain. [43]. The spectra of the SF/B films are significantly influenced by the SF content, indicating that the SF is a major contributor to UV shielding in the UVB and UVA regions. This result indicates that the SF within the SF/B samples maintains its conformation and that B does not alter the SF spectrum.

Conventional methods, such as differential scanning calorimetry (DSC), used to measure glass transition in bulk systems, lack the sensitivity needed to detect this transition in films [44]. Absorption spectroscopy can detect transitions between various electronic states of samples in their absorption region [45]. Alterations in the absorption spectrum result from the molecular-scale reorganization of polymers as the latter are warmed above the T_g_. To determine the T_g_ of the films, we performed UV absorption spectroscopy while increasing the temperature from 25 °C to 100 °C. The deviation metric as a function of temperature is reported in Figure 3b.

The B and SF/B 10/90 films (Figure 3b) exhibited bi-linear curve fitting, with T_g_ at around 80 °C. In accordance with the DSC thermograms reported below, the deviation metric revealed, for the SF and SF/B 50/50 films, the presence of a pre-transition stage at lower temperatures (Figure 3b). In particular, for the SF/B 50/50 film, we observed a transition in the deviation metric at ≈50 °C and the T_g_ at 72 °C. For the SF films, a smoother pre-transition at ≈60 °C was recorded, with the glass transition occurring at 80 °C. The occurrence of transitions recorded before the Tg can be ascribed to the strong water absorption capacity of the Ca^2+^ ions dispersed in the SF films. Water adsorbed on the Ca^2+^ ions forms a hydrated shell that interacts with the amide or hydroxyl groups in the silk proteins. The hydration of Ca^2+^ triggers the average distance of silk chains, thus governing their mobility. Regarding the T_g_ values measured for the SF, these data were consistent with the literature [46,47,48], which reports a T_g_ of 178 °C for dry fibroin films, which, in the presence of bound water, is reduced to 80 °C.

**Figure 4:**
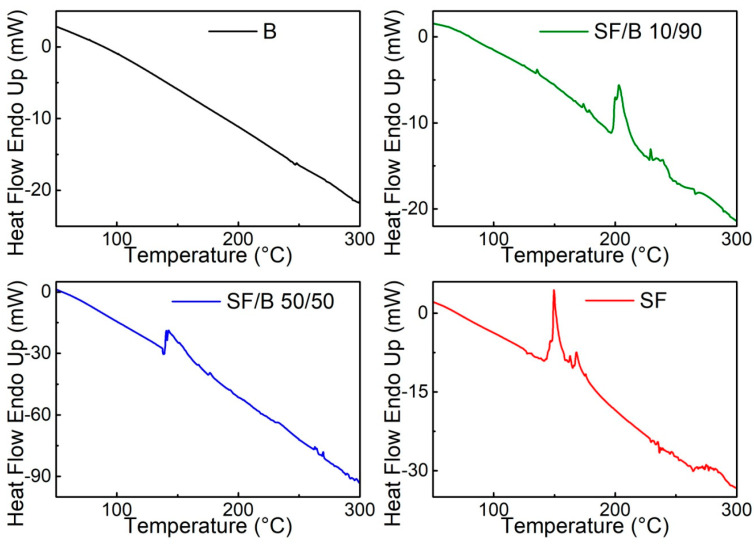
DSC thermograms of the B, SF/B 10/90, SF/B 50/50, and SF films, respectively.

The DSC measurements revealed an endothermic peak between 100 °C and 250 °C for the samples containing SF (Figure 4). This peak was derived from water removal [47]. In particular, the endothermic peak at about 150 °C observed in the SF film was likely related to the evaporation of bound water [48]. This peak was shifted to a higher temperature (e.g., 200 °C) in the SF/B 10/90 sample, suggesting that the higher β-sheet content induced a stronger interaction between fibroin and the water molecules [49]. In the temperature range between 200 °C and 300 °C, another endothermic peak appeared to be caused by the degradation of silk I and silk II [49].

**Figure 5:**
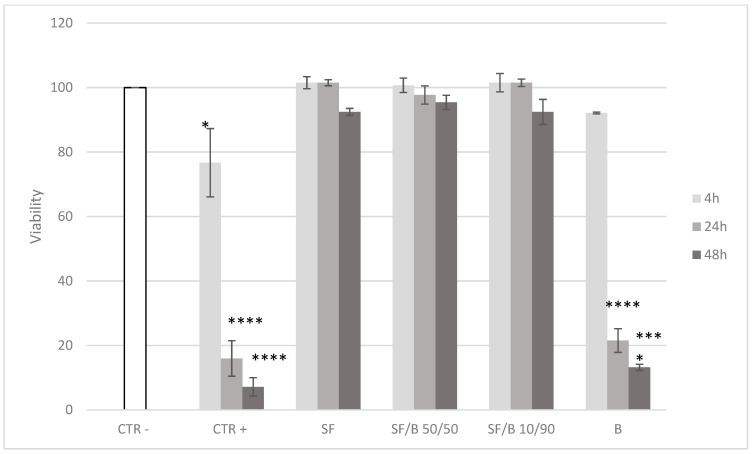
MTT assay on HaCaT cells after 4, 24, and 48 h of 220 s irradiation. * *p* < 0.05, *** *p* < 0.001, and **** *p* < 0.0001.

As in Figure 3a, where the SF showed strong UV absorbance even at the lowest content, these results indicate that the SF/B films are the best candidates for shielding human skin cells from UV radiation. Using an MTT assay, we tested HaCat cell viability after 220 s of irradiation with a cover (CTR−), and we set this as 100% (Figure 5, in white). At the same time, we verified the viability without any type of protection (CTR+), and we observed a gradual decrease just after 4 h from irradiation (*p* < 0.05) and a strong worsening after 24 h and 48 h (*p* < 0.0001). This effect was probably due to nuclear DNA damage and reactive oxygen species production, such as H_2_O_2_, leading to cellular necrosis. HaCat cells covered with the SF (100%) showed a viability comparable to that of CTR−, meaning that no cytotoxic effect was registered. The same results were obtained with SF/B 50/50 and SF/B 10/90 used as a cover, with a cell viability always over 92%, even after 48h from irradiation. On the contrary, when we used B as a lid, the cell viability decreased slightly after 4 h (92.1%) and considerably after 24 h and 48 h by, respectively, 21.5% and 13.2%, both comparable to that of CTR+. The cell viability remained similar to that of CTR− in the presence of SF, even in low concentrations. This means that only SF can suppress biochemical UV-induced cell damage due to its UV-blocking activity.

## 4. Conclusions

In this study, we obtain a hydrophilic, insoluble film with UV-shielding properties by mixing 10 wt% redissolved fibers of silk and bamboo in formic acid. The results suggest that the 10 wt% of SF embedded in the B matrix improved the elastic modulus and strain at break of bamboo by ≈10%, retaining its conformational structure. Moreover, the combination of SF and B acted as a UV-blocking filter, preserving the viability of human keratinocyte HaCaT cells under exposure to the UV component of the solar spectrum. Because of the easy processability of the solution, these findings pave the way for the formulation of environmentally sustainable sunscreens, which may be expanded to wound-healing management.

## Data Availability

Data are contained within the article.
